# Dual Antiretroviral Therapy—All Quiet Beneath the Surface?

**DOI:** 10.3389/fimmu.2021.637910

**Published:** 2021-02-12

**Authors:** Berend J. van Welzen, Patrick G. A. Oomen, Andy I. M. Hoepelman

**Affiliations:** Department of Internal Medicine and Infectious Diseases, University Medical Centre Utrecht, Utrecht, Netherlands

**Keywords:** HIV, antiretroviral therapy, immune activation, dual therapy, cART

## Abstract

Infection with the human immunodeficiency virus (HIV) is characterized by progressive depletion of CD4+ lymphocytes cells as a result of chronic immune activation. Next to the decreases in the number of CD4+ cells which leads to opportunistic infections, HIV-related immune activation is associated with several prevalent comorbidities in the HIV-positive population such as cardiovascular and bone disease. Traditionally, combination antiretroviral therapy (cART) consists of three drugs with activity against HIV and is highly effective in diminishing the degree of immune activation. Over the years, questions were raised whether virological suppression could also be achieved with fewer antiretroviral drugs, i.e., dual- or even monotherapy. This is an intriguing question considering the fact that antiretroviral drugs should be used lifelong and their use could also induce cardiovascular and bone disease. Therefore, the equilibrium between drug-induced toxicity and immune activation related comorbidity is delicate. Recently, two large clinical trials evaluating two-drug cART showed non-inferiority with respect to virological outcomes when compared to triple-drug regimens. This led to adoption of dual antiretroviral therapy in current HIV treatment guidelines. However, it is largely unknown whether dual therapy is also able to suppress immune activation to the same degree as triple therapy. This poses a risk for an imbalance in the delicate equilibrium. This mini review gives an overview of the current available evidence concerning immune activation in the setting of cART with less than three antiretroviral drugs.

## Introduction

In 1983, a group of French virologists identified a T-lymphotropic retrovirus—now called the human immunodeficiency virus (HIV)—as causative agent of the acquired immunodeficiency syndrome (AIDS) (1). The clinical picture of AIDS is characterized by opportunistic infections such as pneumocystis jirovecii pneumonia and candida esophagitis (2). These opportunistic infections are the result of a severe depletion of CD4+ lymphocytes, which are central mediators of immune response, coordinating both cellular and humoral responses against infections (3).

Although HIV uses the CD4 receptor to gain access to target cells, the depletion of CD4+ lymphocytes is only partly due to a direct cytolytic effect of HIV (4). The current leading hypothesis states that chronic HIV infection is accompanied by a hyperactive inflammatory state in which there is an increased turnover of activated naïve T-cells, eventually leading to T-cell depletion by means of apoptosis (5, 6). Immune activation is driven by both the HIV viremia and bacterial translocation from the gut (7, 8) and is associated with numerous comorbidities in HIV-positive patients (9–11). Therefore, immune activation is not only considered to be a predictor for the risk for progression to AIDS but also an important cause of HIV-related comorbidity (12, 13).

Till the end of 1995, the nucleoside reverse transcriptase inhibitors (NRTIs) were the only available antiretroviral agents – targeting reverse transcriptase, an enzyme essential for HIV replication (14). Unfortunately, NRTI mono- or dual therapy had only temporary effects due to rapid resistance development and virological failure (15). However, the perspective for people living with HIV changed dramatically as result of the introduction of a new class of drugs: the protease inhibitors (PIs) combined with a pharmacological booster (16). Combination antiretroviral therapy (cART)—drug regimens consisting of multiple antiretroviral classes—diminished the risk of resistance development and led to an spectacular increase in life expectancy (17). Over the years, the development of antiretroviral drugs took off and several other third drug (“anchors”) classes—such as the non-nucleoside reverse transcriptase inhibitors (NNRTIs) and integrase strand transfer inhibitors (INSTIs) —were introduced (18, 19). Nowadays, triple antiretroviral therapy is highly successful with most patients reaching the main treatment goal of an “undetectable” viral load—defined as <50 copies/ml of HIV RNA when measured by polymerase chain reaction—and with the mortality risk declining (20, 21). Current immunoassays however, due to improvement of sensitivity, are able to detect viral loads that are below 50 copies/ml but can still be quantified: so-called “residual viremia.” A small group of patients—“elite controllers”—are able to maintain an undetectable viral load in absence of antiretroviral drugs (22). However, these patients display significant immune activation when compared to HIV-negative controls (23, 24) and this is linked to an increased risk for cardiovascular disease in these patients (25). These findings emphasize the importance of immune activation in the pathophysiology of HIV-related comorbidity. In the modern antiretroviral era, there is no role for in depth monitoring of immune activation as these markers are generally considered to reduce simultaneously with the viral load, albeit they do not show complete normalization (26).

**Figure 1:**
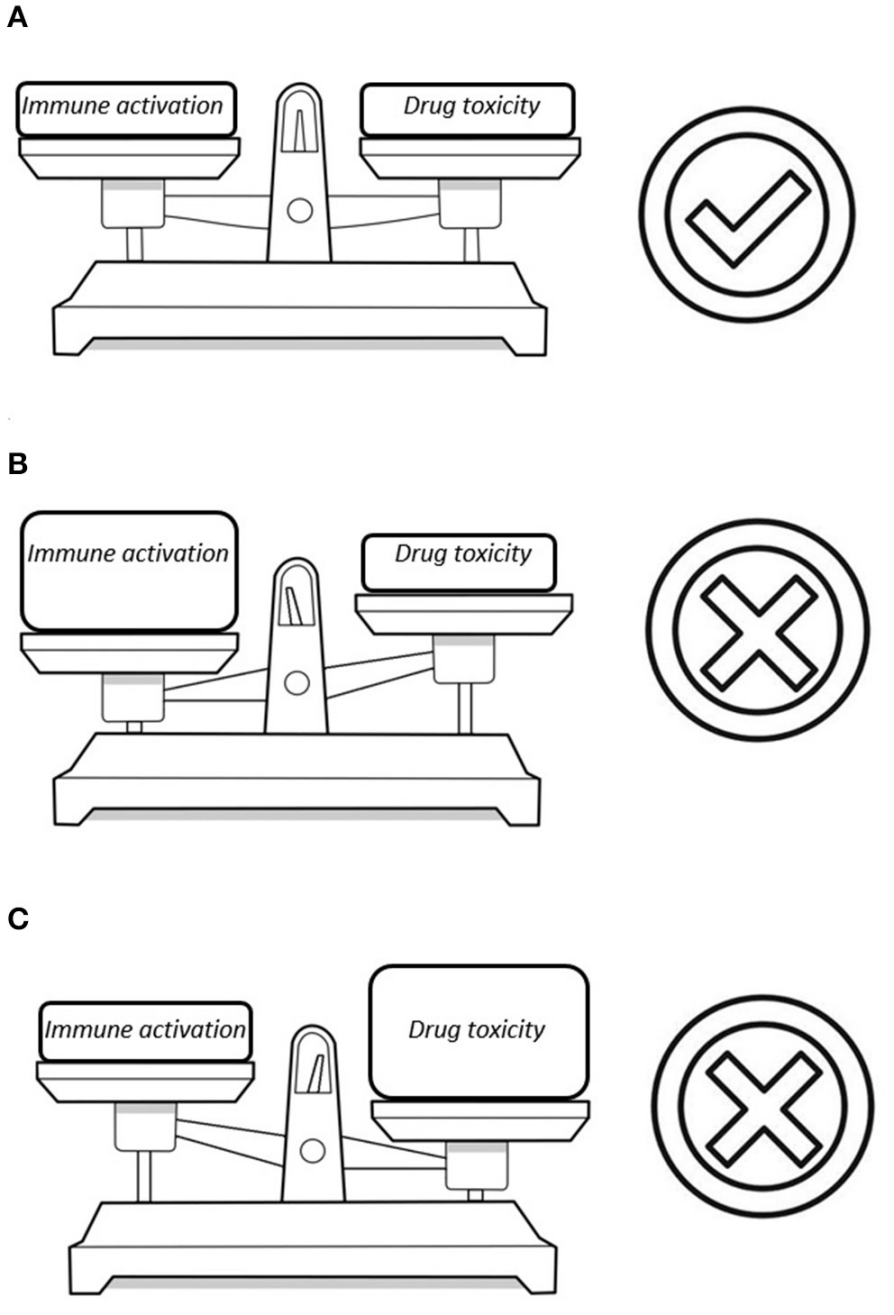
Three possible scenarios in the equilibrium between drug toxicity and damage from HIV-related immune activation: **(A)** A perfect balance between these factors with the smallest possible risk for comorbidity. **(B)** The reduction in the number of antiretroviral drugs diminishes the risk for drug toxicity but a flare in immune activation could lead to HIV-associated comorbidity. **(C)** Multiple antiretroviral drugs are able to fully suppress the virus but this poses a significant risk for cART-associated toxicity.

In the recent years, questions were raised whether there is a need to hold on to the mantra that cART should always consist of three antiretroviral drugs (27). Indeed, the current available agents have high genetic barriers for resistance and the life-long use of multiple drugs could lead to long-term toxicity. Numerous studies evaluated the efficacy of mono- or dual antiretroviral therapy (28–36) and some of these two-drug regimens gained ground in the current treatment guidelines (37, 38). However, there are concerns as to whether the two-drug regimens suppress the degree of HIV-related immune activation enough (39). A rebound in immune activation which occurs beneath the surface despite virological suppression could be harmful. In the end, the development of comorbidity in HIV is the net result of potential harmful effects of antiretroviral drugs vs. the degree in which these drugs suppress the virus and the related immune activation (Figure 1). Therefore, any change in the current standard of care might lead to disruption of this equilibrium. In this mini review, we will discuss the best current available data on immune activation in non-traditional cART regimens.

## Immunological Markers in HIV-Infection

The test battery for HIV-related immune activation is extending ever since the recognition of the hyperactive inflammatory status. The available markers can be divided into soluble and cellular markers for inflammation and immune activation, with some being more readily available than others (40) (Table 1).

**Table 1:** An overview of the most important soluble and cellular markers for HIV-associated immune activation that are reported in current literature.

**Markers**	**Biological and clinical characteristics**	**Ref**.
**Soluble markers**		
Tumor necrosis factor α	- Produced by macrophages and T-cells - Used for cell signaling and cytokine stimulation - Associated with disease progression	47
Interferon-γ (IFN-γ)	- Produced by T-helper cells, CD8+ lymphocytes and NK cells - Induction of several pro-inflammatory cytokines and anti-viral characteristics - Especially active during acute HIV infection	52
Interleukin-6	- Released by monocytes and macrophages - Elevated during chronic stage of infection - Associated with disease progression, especially CVD	46
D-dimer	- Fibrin degradation product -Associated with disease progression, especially CVD	56
Soluble CD14	- Marker of monocyte activation and indirect marker of microbial translocation - Associated with disease progression	58
LPS	- Endotoxin, a marker for microbial translocation - Associated with disease progression	50
Bacterial 16s DNA	- Marker for microbial translocation - Prognostic value in HIV is unknown	50
Soluble CD27	- Marker of T-cel activation - Rapid increase in case of viral rebound	53
Soluble CD40 ligand	- Marker for platelet activation - Implicated to contribute to innate and adaptive immune dysfunction - Prognostic value in HIV is unknown	54
**Cellular markers**		
HLA-DR+	- MHC class II receptor on CD4+ and CD8+ lymphocytes - Upregulated in response to signaling and being a marker for T-cell activation	60
CD38+	- Glycoprotein expressed on lymphocytes and macrophages - Upregulation mediated by IFN- γ and LPS - Considered as a T cell activation marker	61
Ki67+	- Nuclear antigen being a marker for cell proliferation. Present in all cells during mitosis, including T lymphocytes	7
PD-1 co-stimulatory receptor	- Regulating T-cell response - High levels are considered to be result of T-cell exhaustion	63
Annexin-V+	- Marker for apoptosis	62

*For parameters predictive of disease progression, it is not further specified whether this includes declining CD4+ cell counts or clinical AIDS-defining events. CVD, cardiovascular disease; LPS, lipopolysaccharide; Ref, reference; PD-1, programmed death-1*.

The soluble markers are easy to measure in a large number of test facilities and can subdivided into markers of inflammation, coagulation and microbial translocation. The most commonly used inflammation markers include high-sensitivity c-reactive protein (hs-CRP) and plasma interleukin-6 (IL-6), both considered to be extremely sensitive for systemic inflammation (41, 42) and associated with HIV-related mortality (43–46). Other soluble markers include tumor necrosis factor alpha (TNF-α), interferon-γ, neopterin, mitochondrial DNA (mtDNA), β2-microglobulin, soluble CD27, and soluble CD40 ligand (47–54). The latter two are markers of T-cell activation. The main example for coagulation markers is D-dimer, which levels increase in several pro-inflammatory states and high levels being associated with cardiovascular disease (55, 56). The last group of soluble markers are surrogates of microbial translocation. These include bacterial lipopolysaccharide (LPS)—present in gram-negative bacteria—and bacterial DNA (16s ribosomal RNA subunit) (57). In addition, plasma soluble CD14 (sCD14) and soluble CD163 (sCD163)—products of monocyte activation—are also considered to be a markers for impaired mucosal integrity (58). None of these markers are exclusively found in the setting of HIV-infection (59).

Although the soluble markers can be assessed relatively easy, their reflection of inflammation and immune activation is considered to be less specific than the cellular activation markers in the setting of HIV (40). Assessing cellular markers is more labor-intensive, requiring the isolation of peripheral blood mononuclear cells and performing flow cytometry. For the cellular activity, some well-defined markers are available: CD38+/HLA-DR+ expression on lymphocytes for T-cell activation (60, 61), Ki-67 positivity for proliferation (7), annexin-V for apoptosis (62) and programmed-death-1 co-stimulatory receptor for T-cell exhaustion (63). The CD4+ lymphocyte counts and CD4/CD8 ratio are more readily available but these changes occur more slowly and are therefore kept out of this review (64).

## Residual Immune Activation During Triple-Drug Therapy

The initiation of cART results in fast virological suppression and significant reduction in immune activation in most patients, subsequently leading to CD4+ cell recovery (65). However, antiretroviral therapy does not normalize the HIV-induced inflammatory response with some residual immune activation persisting (66). Studies describing the effect of cART on the soluble markers report inconsistent outcomes (43, 67, 68), but especially the degree of T-cell activation rarely normalizes (69).

The clinical impact of this residual immune activation is largely unknown but, for example, the higher incidence of cardiovascular disease among HIV-positive individuals despite cART and the elite controllers implies clinical significance. The reason for residual immune activation in the setting of virological suppression has not been fully elucidated, but it is suggested that low-grade HIV replication in certain anatomical or cellular compartments is the main driver (70). These “sanctuary sites” are compartments, such as the central nervous system (CNS), gastrointestinal tract and lymph nodes, where cART reaches insufficient drug levels to completely suppress local viral replication and subsequent low-grade inflammation. The variable—and often suboptimal—drug penetration in lymph nodes (71), mucosal tissues (72) and the CNS (73) have been demonstrated in several papers. Besides these sites, persisting microbial translocation and the presence of viral coinfections are associated with persistent immune activation (74, 75). There is no consistent evidence that favors one anchor over another with respect to the degree of immune activation (76–78). Studies evaluating whether therapy intensification with additional anchors results in further suppression of immune activation, are conflicting (79–81).

## Immune Activation and Virological Efficacy in Monotherapy

After the introduction of cART in the mid-nineties, monotherapy for HIV-infection was abandoned because of virological inferiority. However, the idea of antiretroviral monotherapy made a comeback after the introduction of agents with a high antiviral potency and a high genetic barrier for resistance. Such a mono-drug regimen have significant advantages, including less side-effects and pill burden. The hypothesis that one powerful antiretroviral drug would be sufficient to maintain virological suppression, led to several trials comparing the virological efficacy of PI or INSTI monotherapy to traditional three-drug regimens (28, 30–33). Unfortunately, monotherapy with these drugs seem to result in higher rates of virological rebound when compared to cART. Therefore, current guidelines recommend against monotherapy as maintenance therapy in treatment-experienced patients with a undetectable viral load (37, 38). However, from a pathophysiological viewpoint it is interesting to have a closer look at the impact of monotherapy on immune activation markers.

One study that provides an insight in the mechanisms of immune activation rebound was published by BenMarzoek-Hidalgo et al. (82). In their paper, the authors describe the relationship between microbial translocation and viremia with immune activation in 71 patients receiving boosted darunavir monotherapy. In this cohort, only 26% of the patients maintained a viral load below 20 copies/ml, while 16 patients displayed virological failure (2 consecutive HIV-RNA levels exceeding 200 copies/mL). The remaining patients had (transitory) episodes of a detectable viral load during follow-up yet without meeting the criteria for virological failure. Although separate analysis per outcome group found that only patients with virological failure showed an increase in T-cell activation, it became clear that time with viral suppression was inversely correlated with T-cell activation (percentage HLA-DR+-CD38+ lymphocytes in both CD8+ and CD4+ lymphocyte subsets) at a follow-up of 24 months. In this study, there was a clear correlation between the viral load and the percentage of activated CD4+ and CD8+ lymphocytes. In addition, another study showed that intensification with INSTI (raltegravir) to PI monotherapy (either darunavir/ritonavir or lopinavir/ritonavir), resulted in a decline in the degree of residual viremia and a decrease in the percentage of activated CD8+ lymphocytes (83).

There are several studies that evaluated the non-specific soluble markers in highly selected populations (84–86), while other studies evaluated the cellular markers. The smallest study of Merlini et al. did not find a difference in T-cell activation between baseline and after 96 weeks for both patients receiving PI monotherapy with atazanavir (*n* = 18) and those receiving atazanavir-based cART [*n* = 22 (87)]. However, patients on monotherapy were more likely to display increased T-cell apoptosis than patients receiving three drugs. Torres et al. evaluated the markers for monocyte activation in 40 patients receiving PI monotherapy (either lopinavir/ritonavir or darunavir/ritonavir) and 20 patients on PI-based cART for at least 48 weeks and an undetectable viral load (88). This cross-sectional analysis showed that patients on monotherapy display higher levels of monocyte activation—CD14+CD16-CD163+ cells and sCD14 levels—when compared to those receiving standard therapy. The last, most well-designed, study of Petrara et al. described the dynamics of the HIV-1 viral reservoir and T- and B-cell activation markers at 48 and 96 weeks of therapy in patients switched to PI mono-therapy (*n* = 32) and patients continuing PI-based triple therapy (*n* = 32) (89). It should be noted that ten percent of the patients in the monotherapy group experienced virological failure compared to zero patients receiving cART. Furthermore, the authors observed a significant increase of T- and B-cell activation in patients receiving one drug, while these markers remained low in patients on cART.

So the best available evidence suggests that a switch to monotherapy is associated with an increase of T-cell activation and apoptosis markers, while soluble markers data are more inconsistent. These observations seem to be the result of (low-grade) viral rebound. The increased risk for virological failure and the suggestion of a rebound in immune activation, disqualify monotherapy as maintenance therapy.

## Immune Activation in Dual Therapy

Antiretroviral monotherapy is not likely to play a role in the near future, so the current focus is on the effectiveness of dual therapy. In fact, two-drug regimens have already gained a position in current HIV treatment guidelines; in 2018 a single-tablet regimen (STR) consisting of dolutegravir (INSTI) and rilpivirine (NNRTI) was introduced and in 2020 a STR with dolutegravir and lamivudine (NRTI) was registered as a first-line treatment option. Currently, there are several large trials that support the use of these two STRs in clinical practice: SWORD-1&2 (36), GEMINI-1&2 (35), and TANGO study (29).

The SWORD-1&2 studies evaluated the efficacy, safety and tolerability of dolutegravir/rilpivirine as maintenance therapy in patients with an undetectable viral load. Patients were randomized to either dual therapy (*n* = 512) vs. continuing triple-drug therapy (*n* = 516). After 148 weeks, the data showed that dolutegravir/rilpivirine was non-inferior with respect to virological outcomes to triple therapy (90). In the first paper evaluating this regimen, there was a brief mention on the dynamics of the inflammatory and cardiovascular markers in both groups. The authors state there was no consistent pattern of change from baseline to week 48 or differentiation between both groups in the following markers: IL-6, CRP, sCD14, sCD163, and D-dimer. Exact data were not shown and specific T-cell markers were not evaluated. The use of STR dolutegravir/lamivudine for treatment-experienced patients is supported by the TANGO study (29). In this study, 743 patients with an undetectable viral load were enrolled and were randomized to either dolutegravir/lamivudine or a triple drug regimen (two NRTIs as backbone and an anchor from one of major groups). In this study, dual therapy was also found to be non-inferior in maintaining virological suppression compared to triple therapy. In the study cohort, the authors describe a significantly smaller decrease in serum IL-6 levels in patients on dual therapy, but for sCD14 there was an exact opposite trend. The dynamics of D-dimer, hs-CRP and sCD163 were comparable for both groups. In the GEMINI-1&2 studies, it was shown that dolutegravir/lamivudine was virologically non-inferior to INSTI-based cART in treatment-naïve patients, but there were no data on immune activation (35).

As mentioned above, the registration trials briefly addressed the concerns regarding HIV-related immune activation in dual therapy. In general, the results were inconsistent and focused on soluble markers. Fortunately, a few other studies described this issue more extensively although not for the registered treatment regimens. In the study of Concepción Romero-Sánchez et al. 58 patients, having an undetectable viral load for at least 6 months, were switched to a two-drug regimen consisting of a boosted PI and Maraviroc, a HIV entry inhibitor; there was no control group in this study (91). The authors observed no change in β2-microglobuline, sCD40L, sCD14, hsCRP, D-dimer, and mtDNA at 24 (±12) weeks of follow-up when compared to baseline. However, for patients with high baseline levels of β2-microglobuline, sCD40L and hsCRP there was marked decrease at final follow-up. Two other papers evaluated the differences between patients on dual antiretroviral therapy vs. those on triple therapy. Belmonti et al. describe the dynamics of IL-6, CRP, sCD14 and D-dimer from baseline to 48 weeks (92). A switch to dual therapy (*n* = 70 boosted atazanavir plus lamivudine) did not result in a significant changes in the markers mentioned above and did not differ from the markers in patients continuing triple therapy (*n* = 69). In addition, Vallejo et al. published a cross-sectional pilot study evaluating a broad spectrum of inflammation and immune activation biomarkers (interferon-gamma-induced protein 10, hs-CRP, sCD14, D-dimer, interferon-γ, TNF-α and IL-4) in patients on dual therapy vs. those continuing triple therapy (93). The dual therapy group consisted of 13 patients that were evaluated at 24 weeks after switch and 36 patients at 48 weeks, the control group included 26 patients. The authors found the lowest IL-6 and sCD14 levels in the patients on dual therapy for 48 weeks; the other markers were not different from the triple-therapy groups. Other studies worth to mention were performed by Quiros-Roldan et al. and Mussini et al. but these papers reported less commonly used parameters such as CD4/CD8 ratio, platelet-to-lymphocyte and neutrophil-to-lymphocyte ratio (94, 95).

In the studies presented above, the switch from triple to dual therapy is not accompanied with a consistent increase in the soluble inflammatory markers. However, in contrast to the monotherapy studies none of the papers assessed T-cell activation, proliferation or apoptosis markers. At this moment, there is sufficient evidence to support certain two drug regimens as treatment options for HIV in terms of virological efficacy but robust data on effects on immune activation are lacking.

## Conclusions

In this review we presented the current best available evidence on the dynamics in immune activation in non-traditional antiretroviral therapy. We found that the most well-designed studies show that monotherapy is associated with insufficient suppression of T-cell activation when compared to traditional triple therapy; there might be an association with a detectable viral load. Furthermore, we observed that the dynamics of T-cell activation, proliferation and apoptosis do not necessarily follow the trends observed in the soluble markers, confirming earlier observations.

Especially the last finding is of great importance when we have a look at the data presented for the two-drug regimens, which now have become a reasonable option in modern antiretroviral therapy. The fact that the large registration trials for treatment-experienced patients included inflammatory makers as secondary outcomes is laudable; it emphasizes the recognition of the importance of this outcome. In contrast, the founders of these studies missed an excellent opportunity for a thorough assessment of the immune activation markers in dual therapy. In SWORD-1&2 and TANGO, the soluble markers are only briefly mentioned or the authors stay away from firm statements. Furthermore, the studies only included soluble markers but there are no data on T-cell activation. As we learned from the monotherapy data, especially those markers might display abnormalities. The fact that T-cell activation is correlated with a detectable viremia and that the two-drug regimens show virological non-inferiority with the 50 copies/ml threshold, is reassuring. However, as we are not aware of the degree of residual viremia in the two-drug regimens, a negative impact of dual drug therapy cannot be excluded at this moment.

Based on the presented studies, we believe there is insufficient evidence that mono- and dual therapy are non-inferior to triple therapy when it comes to the suppression of HIV-related immune activation. Although dual therapy is an attractive option as it diminishes the life-time exposure to antiretroviral drugs with potential toxicity, the impact of a rebound in immune activation are currently unknown. We need to keep the potential negative impact of cART in an equilibrium with the degree of immune activation, as a misbalance could lead to HIV or cART-related comorbidity. There is a need for well-designed, longitudinal studies with a proper, unbiased patients selection evaluating both the soluble and the cellular immune activation markers. Only such studies can tell us whether everything is quiet beneath the surface in dual therapy.

## Author Contributions

BW and PO: draft of the manuscript and editing. AH: critical review and editing. All authors contributed to the article and approved the submitted version.

## Conflict of Interest

The authors declare that the research was conducted in the absence of any commercial or financial relationships that could be construed as a potential conflict of interest.
